# Factors associated with IPV victimisation of women and perpetration by men in migrant communities of Nepal

**DOI:** 10.1371/journal.pone.0210258

**Published:** 2019-07-30

**Authors:** Nwabisa Shai, Geeta Devi Pradhan, Esnat Chirwa, Ratna Shrestha, Abhina Adhikari, Alice Kerr-Wilson

**Affiliations:** 1 Gender and Health Research Unit, South African Medical Research Council, Pretoria, South Africa; 2 School of Public Health, Faculty of Health Sciences, University of the Witwatersrand, Johannesburg, South Africa; 3 Voluntary Services Overseas (VSO) Nepal, Kathmandu, Nepal; 4 Social Development Direct, London, United Kingdom; Stellenbosch University, SOUTH AFRICA

## Abstract

This paper aims to describe the prevalent forms of intimate partner violence (IPV), and the factors associated with IPV among women and men living in the two migrant communities of Baglung district, Nepal. 357 adult women and men were enrolled following a family model, interviewing young married women with daughter-in-law status in the home, their husbands, and mothers-in-law and fathers-in-laws using an electronic questionnaire. Random effects regression modelling compared men and women, as well as young married women with daughter-in-law status and older women with mothers-in-law with status. 28.6% of women had ever experienced physical and/or sexual violence by an intimate partner compared to 18.2% of men ever perpetrated these forms of violence against their wives. Being older, male controlling behaviour and poor relations with husband increased women’s IPV in their lifetime while perceptions that the mother-in-law is kind were protective. Being ashamed of being unemployed and childhood trauma were associated with men perpetrating IPV in their lifetime. Borrowing money or food increased young married women’s lifetime IPV risk while mother-in-law cruelty and male control increased older married women’s lifetime IPV exposure. Factors associated with IPV in the past year among men were being younger, job seeking, experiences of childhood trauma and depression exposure among men while difficulty accessing money for emergencies, holding inequitable gender attitudes, and depression was associated with women’s increased IPV exposure. Unemployment stress, holding inequitable gender attitudes and mother-in-law kindness were associated with young women’s increased IPV risk and hunger, mother-in-law cruelty and depression with older women’s IPV risk. There is a need to critically challenge harmful social and gender norms by using approaches that are sensitive to young married women’s position and unequal gender relations in the family. IPV prevention interventions need to employ a holistic approach that combines changing social and gender norms and improving socioeconomic conditions of women living in migrant communities.

## Introduction

Nepal is one of the South Asian countries with high levels of violence against women and girls (VAWG). This contributed to the global burden of human rights violations and public health problems. Global research indicates that almost a third of women worldwide have experienced physical and/or sexual intimate partner violence (IPV) in their lifetime [1]. A similar prevalence was found in Nepal, where 32.4% of women had ever experienced IPV [2]. This study was conducted among 4,210 pan-Nepali women of reproductive age (15–49 years) found that 17.5%, 23.4% and 14.7% women reported emotional, physical and sexual abuse/violence by their male partners respectively [2]. Other studies found a 16% prevalence of physical IPV and a 26% prevalence of sexual IPV experiences amongst women [3]. 29% of women had forced first sex [4]. The 2011 Demographic Health Survey reported 47% of women had experience any form of violence against women in their lifetime suggesting IPV and domestic violence are highly prevalent in Nepal [5]. Sexual harassment is a pervasive practice in the work place [6, 7]. Understanding the factors that influence women’s risk of victimisation and men’s perpetration of violence is important for the development of effective violence prevention interventions.

Violence within intimate and domestic relationships is a common phenomenon globally and can be attributed to gender norms that promote male dominance over women and women’s acquiescence to male power. Research has shown that VAWG results from a confluence of the individual, relationship, societal and political factors that are driven by pervasive patriarchal norms that perceive use of violence as an acceptable practice [8]. Nepal is a strong patriarchal society with social and gender based discrimination, and socio–cultural norms and practices that tolerate violence against women and legitimise use of violence against women and girls [9, 10]. In this patriarchal culture, men have supremacy over women and women and daughters are viewed as inferior and subordinate to men, and son-preference is advanced to the detriment of daughters and daughters-in-law [11]. 44% of men agreed that a woman deserves to be beaten [3] while 23% of women and 21% of men believed that husbands beating wives is justified if wives do not cook well, refuse to have sex on demand or argue with them [12].

Being married is also a risk factor in Nepal as ever-married women were more likely to have experienced IPV compared with never-married women. The current spouse is the most common perpetrator of IPV [13], while other studies suggested the mother-in-law is a common instigator of IPV [14, 15]. A culture of silence around women’s experiences of violence persists in part due to perceptions that violence is normal or fear of retaliation by partners or in-laws or accusations by the community [11]. Hence IPV has been described as hidden in its nature, occurrence and impacts resulting in a significant underestimation of the real level of harm caused to those who are victimised [16]. This suggests the context where the violence takes place is important. Nepal also has a religion-based culture which espouses conservative social and gender norms. In Hinduism, marriage is obligatory and sacramental, more than just a simple bond between two individuals but a bond between families and a promise of continuity in patriarchal family lines with deep religious, social, and institutional significance [17]. Marriage practices dictate that young people be limited from choosing their own spouses. Once married, a woman is supposed to devote her life to her husband’s service and taking care of his children [17]. Young married women are expected to live with their mothers-in-law in the early years of marriage in servitude towards and under the mother-in-law’s supervision [14].

Nepali women are also not recognized as productive economic citizens and women’s access to education and employment opportunities is limited. Consequently women in Nepal tend to be socio-economically marginalised and ultimately disempowered in part due to the patriarchal norms which place less value in the education of daughters compared to sons and limit daughters’ destinies only to marriage [18]. The United Nations Development Program has reported a 23.8% poverty rate of Nepali people living below US$1.25/day [19]. In 2014, 83% of 27 million people lived in the rural areas, and agriculture provided 33% of the GDP and women earned 57% less income than men [19]. Women had limited access to productive assets such as land and property, credit and modern avenues of knowledge and information.

Nepal is also one of the Asian countries with a highly mobile population. The Nepali economy is increasingly migration-dependent as large numbers of men migrate internally but mostly outside the border for work. Each day an estimated 1400 Nepalese leave the country for overseas jobs, contributing the a total of 527,000 people migrants working outside the country each year [19]. Most migrants are men, and they are likely to leave their wives back at home with limited means of subsistence. Upon their return home from labour outside the country migrant husbands may struggle to adjust to home life in which gendered expectations on them to provide unchanged, to wives and children with whom they have spent a limited time, and to homes which may have remained resource-poor despite having sent remittances. In some instances, women assume some leadership of the home and returnee migrant husbands may struggle with their role as husband. This dissonance may directly or indirectly be associated with husbands’ perpetration of intimate but evidence of this associated is limited in Nepal. Our formative research highlighted the underlying factors associated with IPV were prevalent, particularly overburdening of young married women with domestic work, servitude towards and dominated by in-laws influenced by patriarchal gender norms [14].

Socioeconomic factors have been associated with women’s exposure to VAWG. Survey results indicated higher socioeconomic status posed risk for some women [20]. Though 77% of married women age 15–49 were employed compared 98% of men, 61% of these women are not paid for their work compared to 12% of men [4]. Those who are employed for cash are more likely than other women to experience physical IPV [4, 21] and access to better livelihoods may increase women’s partners’ resistance to corresponding changes in women’s lives [22]. This demonstrates the need to develop programmes to address both gender violence related factors while strengthening women’s livelihoods at the same [23]. Those who are employed for cash are more likely than other women to experience physical IPV [4, 21, 22].[23]

This paper is conceptualised within a context of migration where young married women reside in poor communities where husbands’ migration for labour is a common source of income and wives are more likely to reside with extended affinal families while husbands migrate abroad for work. There is limited data on IPV among migrant communities in Nepal. However, a combination of factors including young married women’s social positioning in the households, poor relations with the mother-in-law, male control over women, poor socioeconomic conditions and conservative gender attitudes may place women at increased risk of IPV victimisation. Attitudes and practices of male dominance over female partners, adversity in childhood and lack of jobs may increase men’s likelihood of perpetrating violence towards their wives. We sought to determine the prevalence of intimate partner violence focusing on women’s victimisation and men’s perpetration, and examined the factors influencing IPV victimisation and perpetration among women and men enrolled in a research project in migrant communities of Baglung, Nepal.

## Methods

### Study design

This paper presents a baseline cross-sectional quantitative study from the One Community One Family project (OCOF), which seeks to develop a family-oriented behavioural intervention to reduce IPV, change gender norms and increase economic empowerment with a bias towards young married women as the most vulnerable to the structural factors influencing IPV risk within the family. These data were collected in January-2017.

### Setting

This study was conducted in two migrant communities, Bhimapokhara and Resha, in the Baglung district of Nepal where a large proportion of men migrate overseas for labour. These neighbouring communities also share similar cultural contexts such as subsistence farming. Baglung is located in hilly landscapes of the western region of Nepal. It covers 1,784 km^2^ with a population of 268,613 [24]. Young married women play a crucial role in tending to crops and livestock [14]. The research was conducted in 6 of 9 municipal wards in the two communities and these wards and are treated as clusters in this study.

### Participants

A sample of 357 participants from 100 families were enrolled into the study at baseline. 100 women who self-identified as daughters-in-law gave consent to participate, followed by the enrolment of their family members based on a family hierarchy, if present in the home, namely, husband, mother-in-law and father-in-law resulting in a total sample of 100 daughters-in-law, 100 100 mothers-in-law, 79 husbands, and 78 fathers-in-law. In this paper, women are categorised as ‘young married women’ if they self-identified as having daughter-in-law status in their home and were aged 18–35 years, and ‘older married women’ if they were the husbands’ mothers. Ultimately 7 young married women were under the age of 20 years which is the legal age of marriage in Nepal and 4 were under the age of 18 years thus indicating existing cases of child marriage. A local nongovernmental organisation, Bhimapokhara Youth Club, recruited participants after obtaining permission for the study from district level stakeholders and community leaders.

### Questionnaire and interviews

Trained interviewers administered to all participants a one-hour questionnaire stored on Tablets. Male and female questionnaires were adapted from previous studies in Tajikistan, and in South Africa [25], and were translated into Nepali and pre-tested for relevance in the Nepali context.All questionnaires had 213 items each, and covered social and demographic characteristics, gender equitable attitudes, household decision making, depression, childhood trauma, sexual and reproductive health, the status of relations with partners and perceptions about the mothers-in-law (and men were asked about their perceptions of their mothers) and intimate partner violence.

#### Social and demographic characteristics

These covered age, ethnicity, levels of education completed, marital status and years married, frequency of hunger in the past 4 weeks, frequency of borrowing money or food because of not having enough money at home, perceived difficulty accessing a modest amount for medical emergencies (NRP1500 or $15). We also asked about activities to get money in the last 3 months, the amounts earned and saved. Questions on migration asked about ever migrating for labour, doing so in the past year and where. Further questions explored job seeking (Cronbach alpha 0.70), feeling stressed about being unemployed (Cronbach alpha 0.81) [26], feeling ashamed due to being unemployed (Cronbach alpha 0.73) and life satisfaction (Cronbach alpha 0.81).

#### Relationship and gender attitude scales

We asked about husbands’ controlling practices over their wives using a modified (9-item) form of the Sexual Relationship Power Scale (Cronbach alpha 0.53) [27],and women’s perceptions about mother-in-law and daughter-in-law relations (and men about their mothers) (Cronbach alpha 0.81), and perceptions about relations between husband and wife (Cronbach alpha 0.74). Community and personal gender norms and attitudes [28], were measured on a 23-item scale with a 4-point Likert response from Strongly Agree to Strongly Disagree (Cronbach’s alpha 0.76).

#### Trauma and mental health

Adverse childhood experiences were measured on a modified version of the short form of the Childhood Trauma Questionnaire [29]. It covered emotional neglect, emotional abuse, physical neglect/hardship, physical abuse, but not sexual abuse (Cronbach’s alpha 0.67). Mental health was measured using the Centre for Epidemiologic Studies Depression Scale (CES-D) scale to assess depressive symptomatology.

#### Intimate partner violence

For the analysis we explored two primary outcomes, lifetime intimate partner violence and intimate partner violence in the past year. The violence questions were adapted for the Nepali context from the WHO multi country study [30] and the Men’s Health study in South Africa [31]. Five items were used to measure lifetime perpetration (men) or experience (women) of physical violence and similar number of items were used to measure physical violence in the past year. Three items were used to measure lifetime or past year sexual violence perpetration or experience, and a total of 7 items were used to measure emotional violence. For each type item, the responses were coded as 0 = never, 1 = once, 2 = few times and 3 = many times. We then derived a dichotomous outcome for experience or perpetration depending on whether a participant had experienced/perpetrated any form of violence or not. This was done for both lifetime and past year measures. This was done for both lifetime and past year measures. We asked men about ever perpetrating physical and sexual violence on their wives and women about ever experiencing it. We further explored perpetration and experiences of emotional, physical and sexual intimate partner violence in the past year.

### Ethics

The study received ethics approval from the South African Medical Research Council and the Nepal Heath Research Council. Each participant signed informed consent prior to completion of the questionnaire. The purpose of the study was explained, and participants allowed to ask questions and responses to those questions were provided. Participants were assigned a unique identification code which was stored on the questionnaire. Each interview was conducted in private to ensure interviews could not be overheard by others and confidentiality was assured. Participants were given information on available psychosocial services in the community. A packet of seasonal vegetable seeds was provided to each participant to compensate for their time.

### Data analysis

The analysis was conducted using Stata 14. In all analysis procedures, we considered the multistage sampling design of the survey, with stratification by district and the enumeration areas being the lowest level cluster. Descriptive statistics were presented as mean with standard deviation (SD) or as frequencies with percentages (%). Bivariate relationships between IPV perpetration/victimisation and attitudinal, behavioural and demographic variables were examined using the Pearson’s Chi-Square test for categorical variables and t-tests for continuous variables.

All factors associated with IPV perpetration/victimisation in the bivariate analysis (p-value <0.10), were considered for the multivariate model, and adjusted for age of participant. The analysis considered variables that fitted with the conceptual framework for exposures to VAWG and these included the mother-in-law and daughter-in-law relationship, relationship conflict, childhood adversity and poverty. To account for clustering by district, standard errors of the estimates were estimated using the clustered robust method. Our criteria for significant risk factors in final multivariate model was the p-value of <0.05.

## Results

357 respondents were recruited, 157 were men and 200 women. Table 1 shows the social and demographic factors as well as gender norms and attitudes, health, behaviour and relationship factors. Research participants were aged 16–83 years, and men were older than women with a mean age of 46.3 years compared to 42.8 years. Young married women averaged 27.9 years of age while older women had an average age of 57.7 years, and young married men had a mean age of 31.5 years while older men had a mean of 61.4 years. All the women participating in the study were currently or previously married and all those previously married were widowers. Only 4 men had never married. The mean age at marriage was 17.1 years for women versus 20.8 years for men. 52% of women had currently lived with husbands’ families while 43% lived only with their husbands and children.

**Table 1:** Social and demographic background of men and women.

	Male (n = 157)		Female(n = 200)		
Variable	n/mean	%/SD	n/mean	%/SD	p-value
Age—16–83 range‡	46.3	18.1	42.8	17.4	0.063
Mean age of Young Married persons‡	31.5	8.1	27.9	7.5	
Mean age of Older persons‡	61.4	11.1	57.7	10.2	
Ethnicity:					
Dalit	18	11.5	27	13.5	0.897
Janajati	71	45.2	89	44.5	
Chhetri	48	30.6	56	28.0	
Brahmin	19	12.1	25	12.5	
Other	1	0.64	3	1.5	
Levels of education passed:					
No education	41	26.1	106	53.0	<0.001
Primary	32	20.4	18	9.0	
Secondary	36	22.9	33	16.5	
SLC Plus (School leaving)	48	30.6	43	21.5	
Age at first marriage ‡	20.8	4.1	17.1	3.24	<0.001
Marital Status					
Currently Married	151	96.2	186	93.0	0.003
Previously Married	2	1.27	14	7.0	
Never Married	4	2.55	0	0.0	
Number of years married ‡	25.8	18.1	25.2	17.8	
Currently living arrangements					
With partner and children	65	42.5	86	43.0	<0.001
With partner's family	1	0.65	103	51.5	
With partner's and natal family	80	52.3	0	0.0	
Alone with children	2	1.31	11	5.5	
With natal family	5	3.27	0	0.0	
Engaged in an activity to get income	90	57.3	43	21.5	<0.001
Ever migrated for work	108	68.8	1	0.5	<0.001
Migrated for work in last 12 months (of those that ever migrated)	90	83.5	0	0	
Difficulty getting money in emergencies	19	12.1	57	28.5	<0.001
Borrowed money or food in the past month	86	54.8	110	55.0	0.97
Hunger score ‡	3.09	0.4	3.34	0.8	0.001
Seeking jobs or doing things for income scale ‡	10.2	3.3	8.8	2.5	<0.001
Unemployment stress scale ‡	9.26	2.0	9.68	2.1	0.06
Ashamed due to unemployment scale ‡	8.67	1.6	9.11	1.7	0.01
Community gender norms and attitudes ‡	47.87	2.6	52.77	4.9	<0.001
Individual gender norms and attitudes ‡	44.08	3.3	45.02	6.4	0.092
Depression scale ‡	9.9	5.3	16.9	9.9	<0.001
Suicidality	2	1.3	39	19.5	<0.001
Childhood trauma score ‡	15.9	2.8	15.2	2.4	0.010
Age of the Partner ‡	42.5	16.9	46.3	17.7	0.03
Relations with one's spouse ‡	15.8	2.6	11.3	1.8	0.774
Mother/Mother-in-law is cruel score ‡	6.4	1.2	6.7	2.4	0.02
Mother/Mother-in-law is kind score ‡	9.1	1.6	8.0	2.8	<0.001
Frequently quarrelling with spouse	42	27.3		41.7	0.009
Relationship control by husband ‡	17.4	0.9	17.5	1.8	0.25
Husbands ever drank alcohol ¥	104	66.2	89	44.7	
Husband drinking in past 12 months ¥	86	82.7			
Involvement in decision-making ‡	11.7	1.4	13.1	2.5	<0.001

‡: Continuous measures summarised by mean and standard deviations. All categorical variables have been summarized using frequencies and percentages.

¥: For men, summary represents their own alcohol use.

The results showed that women reported significantly higher food insecurity compared to men. The hunger score showed a higher mean for women at 3.34 compared to 3.09 for men. More men found it easy to borrow money for emergencies compared with women (88% vs 72%). 55% of both men and women reported having borrowed food or money in last 4 weeks. Many women had never engaged in activities to earn an income in the past 3 months; 22% of women had been involved in some activity to earn income compared to 57% of men.

Two-thirds (69%) of men had ever migrated for work in their lifetime and the majority (84%) had migrated in the past year, but only one woman had ever done so. Almost all the migrant men reportedly sent remittances home (not shown). The results in Table 1 also show that overall men were significantly more likely to look for jobs or do things to create income compared to women although women stressed over finding work and earning income to support their families and felt ashamed to face their families because they are out of work compared to men.

Women held more patriarchal individual gender norms and attitudes compared to men with a mean of 45.0 compared to 44.1 but this difference was not significant. On the participants’ perceptions of community gender norms and attitudes, women reported significantly more patriarchal perceptions of community gender norms and attitudes compared to men (mean of 52.8 versus 47.9).

More women gave a poor or very poor assessment of their overall health compared to men. 25% of women reported having a disability compared to 8% of men. Among women reporting disability, almost half had difficulty in walking, climbing, washing, or concentration (results not shown). Women also had a significantly higher mean on the depression scale with a mean of 16.9 versus 9.9 among men. Men reported having had significantly more childhood trauma compared to women (15.9 versus 15.2, p = 0.01).

Women were less likely to report having good relations with their husbands compared to men. Women were significantly more likely to believe their mothers-in-law were cruel compared to men’s perceptions of their mothers (a mean of 6.7 vs 6.4) while men were significantly more likely believe their mothers were kind compared to women’s perceptions of their mothers-in-law (a mean of 9.1 versus 8.0). There was no significant difference among men and women on reporting of husbands being controlling in relationships.

**Fig 1:**
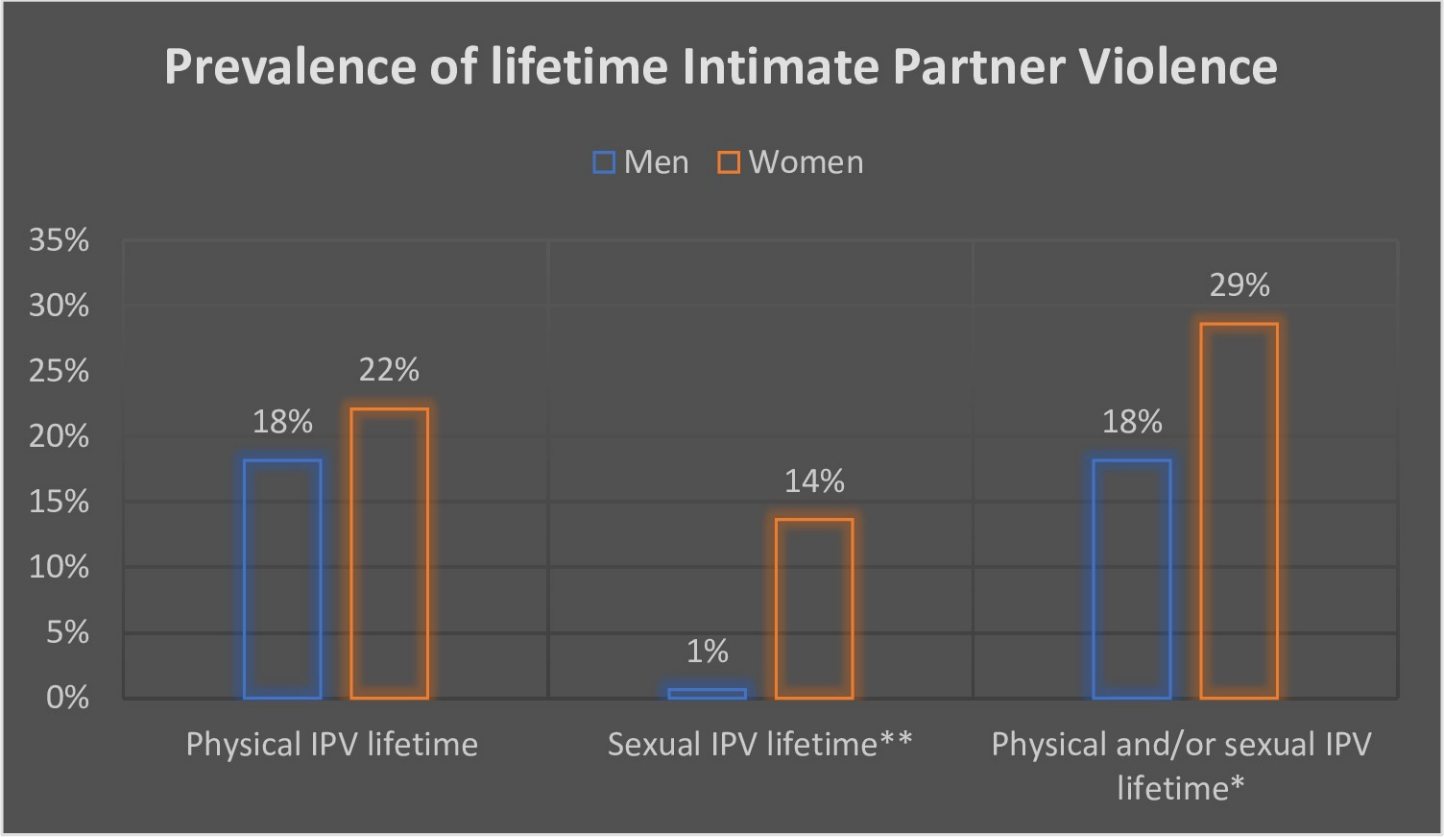
Prevalence of men’s perpetration and women’s experiences of different forms of Intimate Partner Violence in their lifetime.

Twenty two percent (22%) of women reported having ever been physically abused by their husbands while 18% of men had ever physical abused their wives. Significantly one in seven women (14%) had ever been sexually abused by a husband whereas only one man (1%) reported ever doing so to his wife. When these lifetime measures of IPV were combined, 29% of women reported ever experienced physical or sexual violence from by a husband and 19% of men had ever perpetrated physical or sexual violence against their wives. When asked whether they had spoken to anyone outside the family about the violence they were experiencing from their husbands, only 5% of women reported having done so (Fig 1).

**Fig 2:**
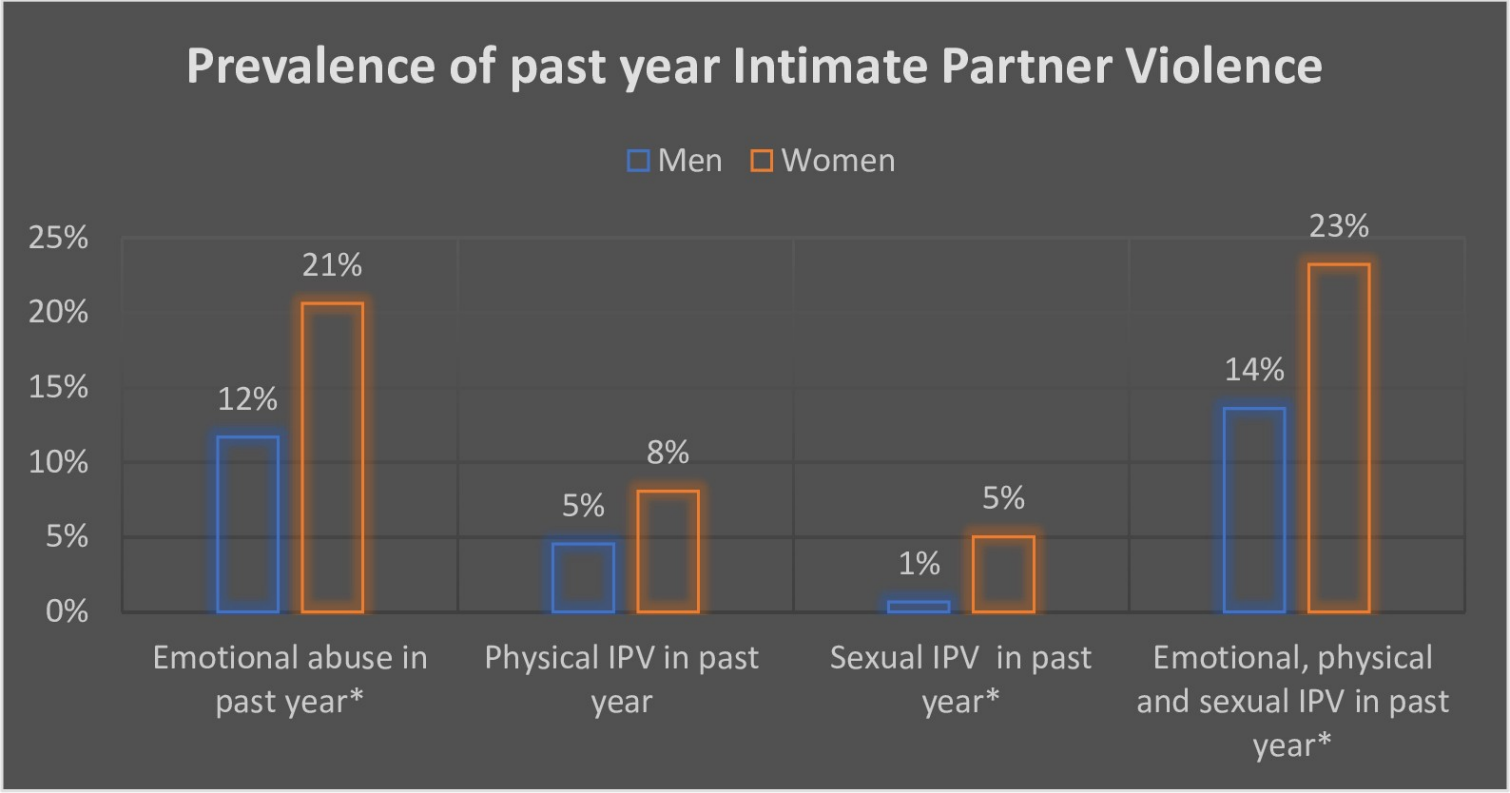
Prevalence of men’s perpetration of and women’s experiences of different forms of Intimate Partner Violence in the past year.

Past year measures indicated that emotional IPV victimisation and perpetration were more common compared to other forms of IPV. Women were significantly more likely to report experiences of emotional IPV by husbands in the past year compared to men reporting perpetration of emotional IPV against wives during the same period (20.6% vs 11.7%, p = 0.03). Women were also twice more likely to experience physical IPV in the past year relative to 4.6% of men having abused their wives in the past year but this difference was not significant. There was significantly more past year sexual victimisation by husbands reported by women to reporting of sexual violence perpetration against wives by men: 5% versus 1%. When all forms of IPV in the past year were combined, women were significantly more likely to have experienced any form of IPV compared to men’s perpetration: 23% of women had reported emotional, physical or sexual IPV in the past year compared to 14% of men (Fig 2).

A detailed account of the bivariate analyses and candidate variables for the random effects multivariate regression analysis are provided as supplementary texts:

S1 Table: Bivariate associations between intimate partner violence in lifetime and social, demographic, gender, relational and other factors by gender and age groups of women.

S2 Table: Bivariate associations between intimate partner violence in the past 12 months and social, demographic, gender, relational and other factors by gender and age groups of women.

Table 2 shows results from regression analyses of associations with lifetime and past year IPV perpetration and victimisation comparing men with women, and women by in-law status. Men who had ever perpetrated physical or sexual IPV were significantly more likely to have experienced shame for being unemployed, and to have experienced adversity in childhood compared to men who had never perpetrated IPV. Among all women in the study, the likelihood of reporting lifetime experiences of physical or sexual IPV was significantly higher if women were older, were controlled by their husbands and had poorer relations with husbands but having perceptions that their mothers-in-law were kind was protective. Analyses of the associations by in-law status showed that borrowing money or food in the past 4 weeks significantly increased the likelihood of young married women with current daughter-in-law status reporting having ever been abused by husbands. Older women with current mother-in-law status who experienced lifetime IPV were more likely to hold perceptions that their mothers-in-law were cruel and to report being controlled by husbands compared to older women who never experienced IPV.

**Table 2:** Multivariate associations of IPV perpetration by men and victimisation of women in lifetime and comparisons among women by in-law status.

	Male perpetration			Women’s victimization			Young women’s victimisation			Older women’s victimization		
Variables	AOR	95% CI	P-value	AOR	95% CI	P-value	AOR	95% CI	P-value	AOR	95% CI	P-value
Being older	1.01	0.97–1.04	0.73	1.02	1.00–1.04	0.03	1.01	0.94–1.09	0.70	0.99	0.95–1.04	0.85
Borrowed money or food	-	-	-	-	-	-	3.16	1.96–5.08	<0.001	-	-	-
Work shame scale	1.40	1.13–1.74	0.002	-	-	-	-	-	-	-	-	-
Mother-in-law is cruel scale	-	-	-	-	-	-	-	-	-	1.63	1.19–2.23	0.003
Mother-in-law is kind scale	-	-	-	0.76	0.65–0.88	<0.001	-	-	-	-	-	-
Relationship control scale	-	-	-	1.26	1.02–1.56	0.03	-	-	-	1.74	1.44–2.10	<0.001
Poor spousal relations scale	-	-	-	1.41	1.17–1.70	<0.001	-	-	-	-	-	-
Childhood trauma scale	1.23	1.18–1.28	<0.001	-	-	-	-	-	-	-	-	-

Table 3 shows regression analyses of associations with past year IPV perpetration. Being younger and work seeking were significantly protective, however, men who perpetrated IPV in the past year had significantly increased odds of reporting childhood trauma and depression compared to those who did not perpetrate violence in the past year. Among women, past year IPV victimisation was associated with increased difficulties to borrow money for emergencies and depression but holding patriarchal individual gender norms and attitudes was protective. Young women with daughter-in-law status had increased odds of experiencing violence by their husbands in the past year if they had unemployment stress while having patriarchal individual gender norms and attitudes and holding perceptions that mothers-in-law were kind were protective. Older women with mother-in-law status were more exposed to violence by their husbands in the past year if they reported a higher hunger score, perceived their mothers-in-law to be cruel and reported depression.

**Table 3:** Multivariate associations of IPV perpetration by men and victimisation of women in the past year with comparison among women by in-law status.

	Male perpetration			Women’s victimization			Young women’s victimization			Older women’s victimization		
Variables	AOR	95% CI	P-value	AOR	95% CI	P-value	AOR	95% CI	P-value	AOR	95% CI	P-value
Being older	0.97	0.95–0.99	0.05	0.99	0.96–1.02	0.64	1.01	0.93–1.10	0.81	0.99	0.94–1.04	0.56
Hunger score	-	-	-	-	-	-	-	-	-	1.77	1.11–2.81	0.02
Difficulty getting money for emergencies	-	-	-	2.76	1.03–7.38	0.04	-	-	-	-	-	-
Work seeking scale	0.85	0.76–0.96	0.008	-	-	-	-	-	-	-	-	-
Unemployment stress scale	-	-	-	-	-	-	1.39	1.18–1.64	<0.001	-	-	-
Individual gender norms and attitudes scale	-	-	-	0.92	0.86–0.98	0.01	0.88	0.84–0.92	<0.001	-	-	-
Mother-in-law is cruel scale	-	-	-	-	-	-	-	-	-	1.94	1.10–3.39	0.02
Mother-in-law is kind scale	-	-	-	-	-	-	0.64	0.54–0.76	<0.001	-	-	-
Childhood trauma scale	1.42	1.24–1.63	<0.001	-	-	-	-	-	-	-	-	-
Depression scale	1.11	1.06–1.16	<0.001	1.05	1.01–1.10	0.01	-	-	-	1.06	1.02–1.11	0.004

## Discussion

The baseline study results from the OCOF project showed that the prevalence of IPV perpetration and victimisation in two migrant communities of Baglung district in Nepal is driven by poor socioeconomic conditions of women, male controlling behaviour over wives, poor relations with husband and mother-in-law, men’s exposure to trauma in childhood and men and women’s experiences of depression at the time of the study.

Almost a third of women had ever experienced physical and/or sexual violence by their husbands while almost one in five men had ever done so to their wives. About one in five women had experienced emotional, physical and/or sexual violence by their husbands in the past year compared to one in seven men who perpetrated these forms of violence against their wives during the same period. However, these figures indicate there was underreporting of IPV in this study. These data on lifetime physical and sexual IPV victimisation are lower compared to the 2011 Demographic Health Survey [32] and other population estimates on emotional IPV [2] or physical or sexual IPV victimisation [5, 33]. Male perpetration of any form of IPV was also dissimilar to the high prevalent reporting observed in other men’s studies in which some men of whom were migrant labourers [34]. Some of the gaps in the lower levels of reporting among women can be attributed to a cultural environment where violence against married women is tolerated [9, 10, 34], and silence about family and violence-related problems is encouraged [11]. Commensurate with this notion, only 4.5% of victimised women in this study had ever informed anyone outside their marital family about the violence experienced at home (results not shown). This lack of disclosure of violence victimisation and perpetration was also observed in the formative qualitative research preceding this baseline study [14]. The early age of marriage among many women in Nepal is another significant contextual factor influencing whether women report on domestic and intimate partner violence. The mean age at which women were married in this study was 17 years which is much younger than the legal marriage age of 20 years in Nepal. This suggests that these women had experienced child marriage. Research shows that women married as children are at a higher risk of IPV [35, 36]. IPV also occurred within a predominantly male migration context where young married women are heavily burdened with domestic work, and their lives are defined by living in servitude towards and are dominated by in-laws [14].

Regression analyses of factors associated with women’s experiences of IPV show women’s poor socioeconomic conditions increased their odds of being exposed to violence by their husbands in their lifetime and in the past year. Borrowing money or food in the past 4 weeks increased young married women’s risk of IPV in their lifetime threefold, their risk for IPV in the past year was twofold from having difficulties accessing money for emergencies, and hunger also influenced older women’s IPV risk in the past year. These results are consistent with other studies that have found that poorer women have increased exposure to IPV [2]. The socioeconomic conditions of men also came to light as men’s shame over being unemployed increased their risk of IPV perpetration while being engaged in job seeking was protective.

These demonstrate clear connections between IPV and socioeconomic factors associated with women and men’s risk of IPV. There is also concurrence with formative research suggesting that women’s lack of access to economic resources influence their IPV risk, and that constructions of masculinity are centred on men’s ability to earn in this setting [14]. The study results also indicated that older women had significantly higher exposure to IPV, thus mirroring the 2011 Demographic Health Survey results [2]. These results support the need for IPV prevention interventions that focus on economic empowerment, particularly of women.

The younger married women were also found to be more likely to experience any form of IPV if they reported having stressed over being unemployed. This measure referred to stress over personal state of unemployment so that it is safe to propose the unemployment stress may be a proxy for women’s lower socioeconomic status. Other scholars have shown that the likelihood of experiencing violence is higher among rural women with low socioeconomic status [5]. However, there are contradicting claims that women who are employed have increased risk for IPV [2, 33]. This suggests that while women may feel pressure due to unemployment, the nature of interpersonal relations with husband and the extended family may be the underlying factors driving the violence. These findings can also be related to the evidence indicating that men who were ashamed due to having no money or jobs were violent towards their wives. An in-depth understanding of women’s socioeconomic aspirations is also required to provide a clear interpretation of these results to inform IPV prevention interventions that can help improve women’s economic conditions while building harmony with husbands who are likely to be unemployed.

Male controlling behaviour also emerged as having significant associations with women’s lifetime exposure to IPV, and this was evident among the older women with mother-in-law status. Men’s control over wives is linked to entrenched patriarchal social norms where women and girls occupy lowly social positions relative to men and boys in Nepali society. In the Nepali context, patriarchal social norms ascribe male household decision-making and economic control as a masculine preserve in families [7], and promote multiple restrictions on the mobility, education and economic participation of women [14]. These resonate Jewkes and colleagues who purported that “violence is a consequence of gender power inequities at both a societal and relationship level, and also serves to reproduce power inequities” [37, 38]. Poor relations with husbands was also associated with women’s exposure to lifetime experiences of IPV thus emphasising that marital discord is a key ingredient in the circumstances leading to violence against married women [1, 11]. Other studies also suggest that IPV thrives in relationships with poor spousal communication [39].

Women holding patriarchal gender norms and attitudes was protective against their exposure to IPV in the past year. This reinforces the role of acquiescent femininity that supports and excuses dominant patriarchal norms that promote male dominance over women and tolerate gender inequalities and violence against women. Other studies show that there are social sanctions applied by husbands, in-laws and the community against women who do not conform to expected gender roles [14, 15]. It is thus plausible that some young women would defend traditional inequitable gender ideologies [40] as older women do so to protect their interests [41]. Support for gender inequitable norms may also be a product of socialisation into marital relations, particularly as some women marry at a much younger age and are immediately placed under the authority of mothers-in-law whose major role is to perpetuate traditional gender norms for the family [14], and who exert expectations of respect towards elders [42]. For example, young bride’s lives in South Asia are governed by subservience to the mother-in-law, but as they get older they are likely to adopt the same unequal power relations with their son’s wives [41]. These results emphasise the connections between gender inequitable norms and the prevalence of VAWG and highlight the need to transform inequitable gender norms and ideologies to prevent VAWG [43].

The data also showed that women who held perceptions that the mother-in-law was kind were protected from experiencing any form of IPV. This may have been an indicator of harmonious relations with the mother-in-law, against the backdrop of many studies that have described the daughter-in-law/mother-in-law relationships as often contentious [15, 35]. The image of the mother-in-law in some Asian studies is one that depicts dominance [44, 45], cruelty [44, 46, 47], and the usual instigators of intimate partner violence [35, 48] and even perpetrator of domestic violence towards daughters-in-law [11, 46]. The role of the mother-in-law in IPV is likely evident as demonstrated in the associations between IPV and poorer relations with husbands among young married women with daughter-in-law status in this study.

Older women in this study were also mothers-in-law in their own families, and their perceptions that the mothers-in-law was cruel increased their lifetime exposure to IPV. Our results concur with other studies in highlighting the dominant femininity among mothers-in-law who exert control over young married women, and can be likened to the notion of complicit femininity whereby women are involved in perpetuating patriarchal gender norms [14, 49–51], which also make violence against women more permissible. Scholars suggest that this is an internalised patriarchal ideology to gain security in old age by building allegiance with the son while alienating the daughter-in-law [41]. These results indicate the need to develop interventions that address the role of the mother-in-law in creating harmony in family relations.

The analysis also indicated that depression was associated with both men and women’s exposure to IPV perpetration and victimisation respectively in the past year. This connection between emotional distress and IPV has been observed elsewhere, and particularly in relation to women’s report of victimisation by male intimate partners [25, 48, 52–55]. The absence of specific associations with young married women in the regression analyses may imply temporality, thus suggesting that depressive symptoms may be a consequence of long-term exposure [11]. IPV prevention efforts developed for the Nepali context need to address the psychosocial impact on all married women.

The men’s results also indicate that experiences of childhood trauma were associated with men’s perpetration of IPV. Other research suggests that having experienced or witnessed violence in childhood is associated with adulthood perpetration of violence [56, 57]. Others have found pathways between co-occurrence and a cycle of abuse and childhood trauma leading to increased risk of perpetration of violence during adulthood and attribute some of this to harsh parenting and the normalisation of violence [58]. This finding suggests the need to address social and gender norms that promote the acceptance of violence at the family and community level and ensure that adequate support is provided to abused women and children, and positive parenting skills are provided at family and community levels.

## Limitations

The study had several limitations. The sample was drawn from a small geographical area in a country that is heavily reliant on migrant labour. This sample may not be fully representative of the spectrum of migrant communities in the country. Due to funding limitations, participants from a few families were enrolled, and as expected in a male migrant labour context, there were fewer men in households. This underpowered the study, resulting in large confidence intervals for some variables and implies that prevalence estimates cannot be generalized across all married women and men in migrant communities in Nepal. Current migration of husbands was not directly measured among women as we understood most families had a male migrant as a member of their family and anticipated very few women would have gone abroad for migrant labour. Some aspects of VAWG were not measured, including domestic violence by mother-in-law and father-in-law. The study design of working with the young married woman’s family, her husband and in-laws introduced limitations that likely contributed to underreporting of IPV victimisation and perpetration, and potentially other variables. This may be the case despite measures to ensure all participants were interviewed separately, in private and not on the same day as the other members of the family. However, the data is sufficient to provide a good understanding of a limited area of study on the factors influencing women’s exposure to and men’s perpetration of IPV, and the role of in-laws in women’s increased risk for IPV in migrant communities in Nepal. Volunteerism may also have introduced biases in the relationship between variables in ways that are difficult to determine. To address some of the limitations of the study, questions were piloted. In all analyses we controlled for age and clustering. Interpretation of data was triangulated by the VSO team engaging in data analysis, reflecting on the Nepali context particularly regarding the economic conditions and patriarchal gender norms and practices in rural migrant communities.

## Conclusion

The study results revealed that poor socioeconomic conditions of women, male controlling behaviour over wives, poor relations with husband and mother-in-law, men’s exposure to trauma in childhood and men and women’s experiences of depression played a critical role in increasing women’s exposure to IPV and men’s perpetration of IPV. This raises the need to critically challenge harmful social and gender norms by using approaches that are sensitive to the vulnerability of young married women and unequal power relations with husbands and mother-in-law at the family level. These programmes should also include social and mental health support services to address recent, and long-term consequences of violence exposure for women and men, and young married women’s maltreatment by mother-in-law. Young married women stressing about unemployment is suggestive of the need to acknowledge their livelihood aspirations in a setting which often undermines their agency. A holistic IPV prevention approach in migrant communities which combines changing social and gender norms, addressing psychosocial needs and improving socioeconomic conditions of women and men while accounting for the limited resources is needed in Nepal. Gender transformative, violence reduction and livelihoods strengthening programmes stand a chance in building harmonious family and couple relations and reducing women’s exposure to violence while improving their economic conditions if implemented together. Such an intervention would need to be relevant to the cultural context, the nature of families and socioeconomic conditions of women in families in Nepal.

## Supporting information

S1 Table Bivariate associations between intimate partner violence in lifetime and social, demographic, gender, relational and other factors by gender and age groups of women. (TIF)

S2 Table Bivariate associations between intimate partner violence in the past 12 months and social, demographic, gender, relational and other factors by gender and age groups of women. (TIF)

S3 Table Men’s data. (CSV)

S4 Table Women’s data. (CSV)

S5 Table Women’s baseline questionnaire. (PDF)

S6 Table Men’s baseline questionnaire. (PDF)

S7 Table List of derived variables. (PDF)

S1 Text (TIF)
