# Supplementary figures and images for: Factors associated with IPV victimisation of women and perpetration by men in migrant communities of Nepal
Source: PLoS One. 2019 Jul 30;14(7):e0210258. doi: 10.1371/journal.pone.0210258 (PMC6667197; doi:10.1371/journal.pone.0210258)

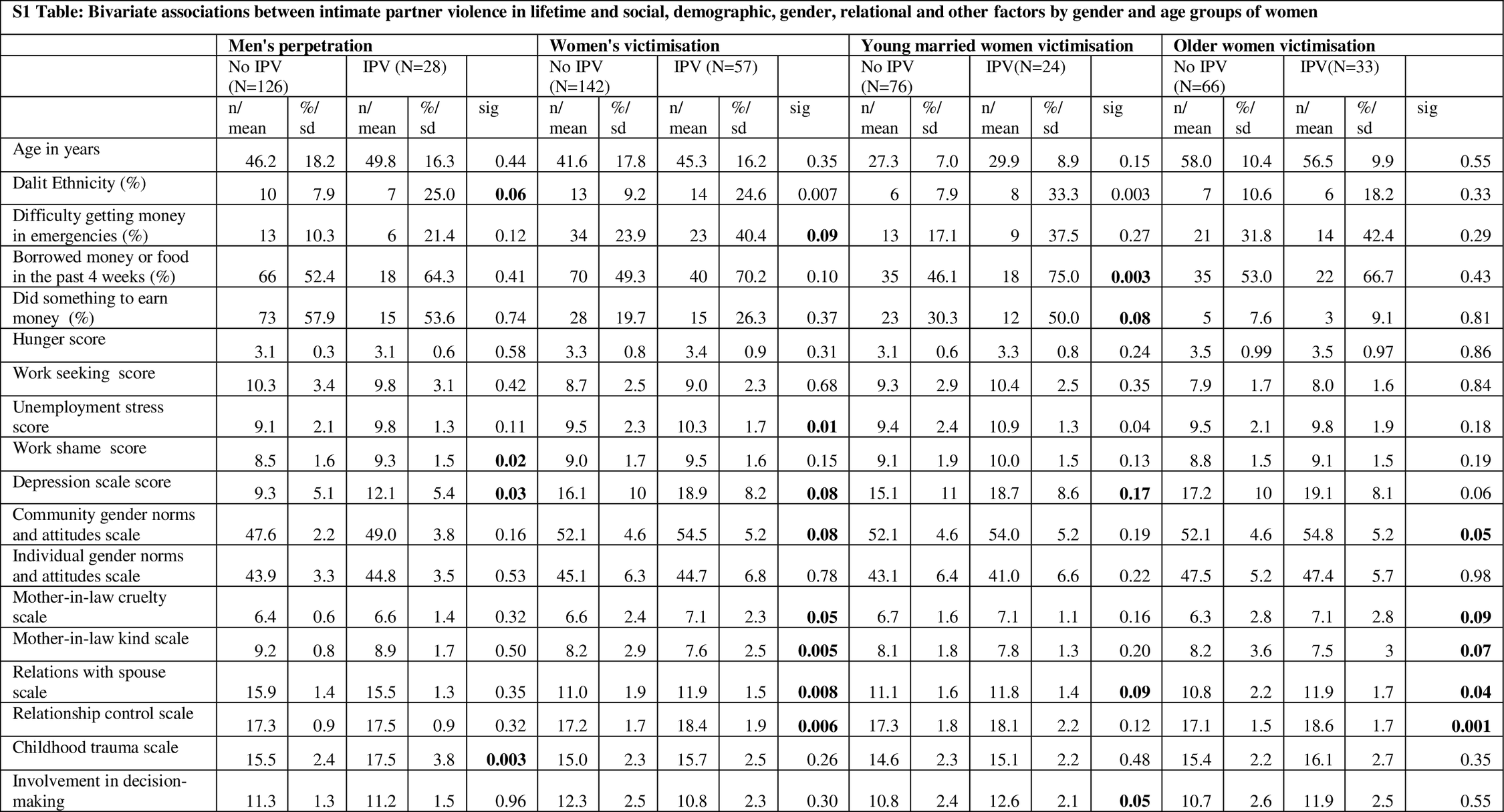

Supplement: S1 Table — (TIF) [file pone.0210258.s001.tif]

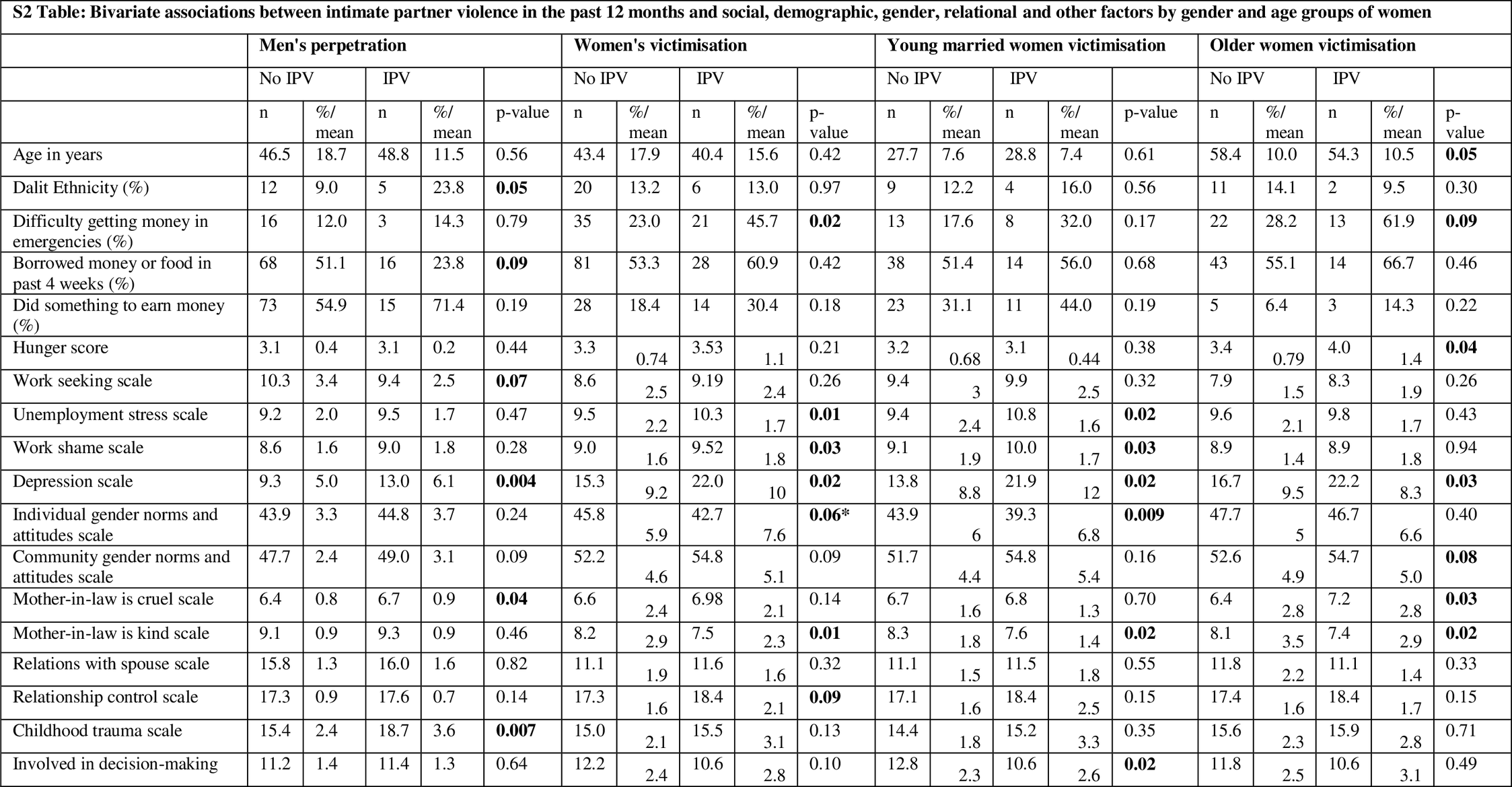

Supplement: S2 Table — (TIF) [file pone.0210258.s002.tif]

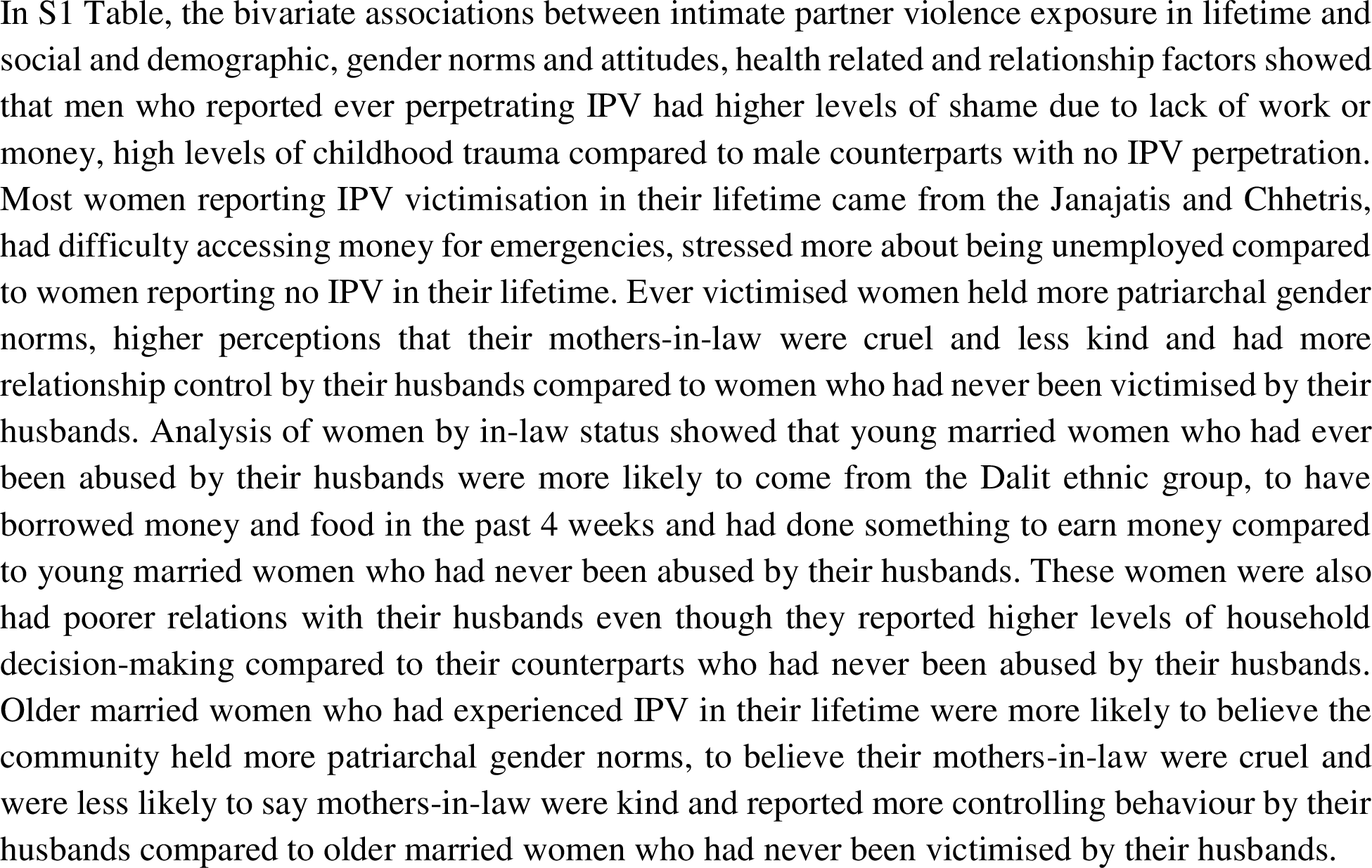

Supplement: S1 Text — (TIF) [file pone.0210258.s008.tif]
