# Supplementary material for: Factors associated with IPV victimisation of women and perpetration by men in migrant communities of Nepal
Source: PLoS One. 2019 Jul 30;14(7):e0210258. doi: 10.1371/journal.pone.0210258 (PMC6667197; doi:10.1371/journal.pone.0210258)
Supplement: S5 Table — (PDF) [file pone.0210258.s005.pdf]

WOMEN'S

QUESTIONNAIRE

BASELINE

COVER

Study ID number: _______________

DATE: ________________

SECTION ONE: BACKGROUND & HOME

The first questions are about yourself, your home and your work situation. Please try and relax, there are no right or wrong answers. Remember that everything you answer will be kept secret.

	QUESTIONS AND FILTERS	CODING CATEGORIES	
101	How old are you?	AGE (YEARS)	
102	What is your caste?	Dalit ....1<br>Janjati ....2<br>Chettri ....3<br>Brahman..4<br>Other 5	
103	Have you lived in this village all your life?	Yes..1<br>No..0	
104	Did you attend school?	Yes..1<br>No..0	If no<br>→ 108
105	What is your highest level of schooling?	Primary (1-4) .... 1<br>Completed primary (5).. 2<br>Lower secondary (6-8) .... 3<br>Secondary (9) .... 4<br>Completed secondary (10) ....5<br>SLC .... 6<br>Intermediate/10+2 .... 7<br>Graduate/Bachelors .... 8<br>Post Graduate/Masters & PhD................................. 9	
106	Have you been to university or attended courses after school?	YES...1<br>NO 0	
107	Do you have you a certificate or diploma from post-school education or training after school?	YES...1<br>NO 0	
108	Are you currently studying?	YES...1<br>NO 0	
109	Are you current married or have you been previously married?	CURRENTLY MARRIED..............................1<br>PREVIOUSLY MARRIED..............................2<br>NEVER MARRIED.......................................3	If 1 go to 110<br><br>If 3, go to no<br>→ 116
109 a	Did your previous marriage end in divorce or were you widowed?	WIDOWED.......................................1<br>DIVORCE...2<br>SEPERATED.......................................3<br>ABANDONED.......................................4<br>HUSBAND DISAPPEARED 5	
110	How long have you been married? (Or how long were you married for?)	[ ] YEARS	
111	Have you only been married to your current (or last) husband or did you marry before that? If so how many times?	CURRENT (PREVIOUS) HUSBAND ONLY.............1<br>MARRIED TWICE..2<br>MARRIED THREE TIMES......................................3<br>MARRIED MORE THAN THREE TIMES..................4	
112	Are you related to your husband?	NO..0<br>YES, COUSINS..1<br>YES, OTHER RELATIVE.......................................2	
113	How old were you when you married (or were first married, if there has been more than one marriage)?	[ ] AGE IN YEARS	

114	Do you live (READ OPTIONS):	ALONE WITH YOUR HUSBAND AND CHILDREN (IF YOU HAVE CHILDREN) ……………………………………1<br>IN SAME HOUSEHOLD WITH YOUR HUSBAND'S FAMILY……………………………………………………2<br>IN SAME HOUSEHOLD WITH YOUR HUSBAND AND YOUR OWN FAMILY…………………………………………3<br>ALONE WITH YOUR CHILDREN…………………………4<br>WITH YOUR FAMILY……………………………………5<br>ALONE……………………………………………………6	

WORK AND MONEY			
116	In the past 4 weeks, how often was there no food to eat of any kind in your house because of a lack of money?	Often……………………………………4<br>Sometimes…………………………………3<br>Rarely……………………………………2<br>Never……………………………………1	
117	In the past 4 weeks how often did you or any member of your household go to sleep hungry because of lack of food?	Often……………………………………4<br>Sometimes…………………………………3<br>Rarely……………………………………2<br>Never……………………………………1	
118	In the past 4 weeks how often did people in your home go without meat due to lack or money or to save money?	OFTEN HAVE NO MEAT……………………1<br>SOMETIMES HAVE NO MEAT…………………2<br>OCCCASIONALLY HAVE NO MEAT……………3<br>ALWAYS HAS MEAT…………………………4<br>VEGETARIAN…………………………………5	
119	In the past 4 weeks how often did people in your home was eating fruit like apple, orange, mango, papaya?	EVERY DAY……………………………………5<br>MORE THAN ONCE A WEEK…………………4<br>ALMOST EVERY WEEK………………………3<br>ONCE OR TWICE IN THE LAST 4 WEEKS ………2<br>NEVER…………………………………………1	
120	In the past 4 weeks how often did you or any of your household go a whole day and night without eating because of lack of food?	Often……………………………………4<br>Sometimes…………………………………3<br>Rarely……………………………………2<br>Never……………………………………1	
121	If you had an emergency at home and needed 1500 Nepal Rupees, how easy would you say it would be to find the money?	VERY DIFFICULT……………………………1<br>SOMEWHAT DIFFICULT………………………2<br>FAIRLY EASY…………………………………3<br>VERY EASY……………………………………4	
122	How often in the past 4 weeks have you had to borrow food or money because you did not have enough?	EVERYDAY ……………………………………5<br>MORE THAN ONCE A WEEK ………………4<br>ALMOST EVERY WEEK ………………………3<br>ONCE OR TWICE IN THE LAST 4 WEEKS……2<br>NEVER …………………………………………1	
123	Have you done anything which has earned money for you or your family in the last 3 months?	YES……………………………………1<br>NO………………………………………0	IF NO<br>➔126
127	Have you ever moved away from home in for work?	YES……………………………………1<br>NO………………………………………0	IF NO<br>→132
129	In the last 12 months have you worked away from home?	YES……………………………………1<br>NO………………………………………0	IF NO<br>→132

132	For the following statements, please tell me how often in the **last 3 months** you have done the following:	NEVER	ONCE	A FEW TIMES	MANY TIMES
	Searched for work	1	2	3	4

	Searched newspapers for jobs	1	2	3	4
	Handed in or sent off an application for work	1	2	3	4
	Offered to work without pay to get experience	1	2	3	4
	Worked without pay to get experience	1	2	3	4
	Developed an idea for a way of earning by selling or making things	1	2	3	4
	Earned money through selling or making things	1	2	3	4
133	The following statements are a series of statements about your current work situation. Please say if you strongly agree, agree, disagree or strongly disagree with these phrases:	STRONGLY DISAGREE	DISAGR EE	AGREE	STRONGLY AGREE
	I am frequently stressed or depressed because of not having enough work	1	2	3	4
	I am frequently stressed or depressed because of not having enough income	1	2	3	4
	I am frequently stressed or depressed because I am not proud of what I do to get money	1	2	3	4
	I am frequently stressed or depressed because I want or have to help my family with money	1	2	3	4
134	The following statements are series of statements about your work situation. Please say if you strongly agree, agree, disagree or strongly disagree with these phrases:	STRONGLY DISAGREE	DISAGR EE	AGREE	STRONGLY AGREE
	I sometimes feel ashamed to face my family because I am out of work.	1	2	3	4
	I spend most of my time out of work or looking for work	1	2	3	4
	I have given up looking for work because I never find any	1	2	3	4
	I am ashamed to see my husband because I don’t have money	1	2	3	4

SECTION 2 ATTITUDES ABOUT RELATIONS BETWEEN MEN AND WOMEN					
The next set of questions are about your views on life and particularly on relations between men and women in society. There are no right or wrong answers – we are just interested in what you think.<br><br>For each of the following statements please say answer whether you strongly agree, agree, disagree or strongly disagree with the following statements:					
201		STRONGLY DISAGREE	DISAGREE	AGREE	STRONGLY AGREE
A	In this community most people think that girls should complete secondary school (to grade 10)	1	2	3	4
B	I think girls in my family should go complete secondary school (to grade 10)	1	2	3	4
C	In this community most people think that girls should continue their education after completing secondary school	1	2	3	4
D	I think girls in my family should continue their education after completing secondary school	1	2	3	4
E	In this community many people think that a wife must ask permission from her husband or his family before going somewhere	1	2	3	4
F	I think the wives in my family must ask permission from her husband or his family before going somewhere	1	2	3	4
G	In this community many people think that wives who live with their husband's parents should have a say in how money is spent	1	2	3	4
H	I think the wives who live with their husband's parents should have a say in how money is spent	1	2	3	4
I	In this community many people think that husbands should allow their wives to do something at home to generate income for the family	1	2	3	4
J	I think the husbands in my family should allow their wives to do something at home to generate income for the family	1	2	3	4
K	In this community many people think that husbands should allow their wives to do something outside the home to generate income for the family	1	2	3	4
L	I think the husbands in my family should allow their wives to do something outside the home to generate income for the family	1	2	3	4
M	In this community many people think that husbands should allow their wives to have a job to contribute to the family budget	1	2	3	4
N	I think the husbands in my family should allow their wives to have a job to contribute to the family budget	1	2	3	4

O	In this community many people think that a husband should be kind and care about the happiness of women in his family	1	2	3	4
P	I think husbands in my family should be kind and care about the happiness of women in the family	1	2	3	4
Q	In this community many people think that wives must always obey their husband	1	2	3	4
R	I think that the wives in my family must always obey their husbands	1	2	3	4
S	In this community many people think that a daughter-in-law must always obey her mother-in-law	1	2	3	4
T	I think that the daughters-in-law in my family must always obey their mother-in-law	1	2	3	4
U	In this community many people think that if a wife does something wrong her husband has the right to punish her	1	2	3	4
V	I think that if a wife in my family does something wrong her husband has the right to punish her	1	2	3	4
W	In this community many people think that a wife of any age who does things that are wrong should be beaten to correct her behavior	1	2	3	4
X	I think that a wife in my family of any age who does things that are wrong should be beaten to correct her behavior	1	2	3	4
Y	In this community many people think that it is acceptable to beat a young wife to teach her how to behave properly	1	2	3	4
Z	I think it is acceptable for a young wife in my family to be beaten to teach her how to behave properly	1	2	3	4
AA	In this community many people think that a woman should tolerate violence in order to keep her family together	1	2	3	4
AB	I think that a wife in my family should tolerate violence in order to keep her family together	1	2	3	4
AC	In this community many people think that a woman should tolerate violence for sake of family honour	1	2	3	4
AD	I think that a wife in my family should tolerate violence for sake of family honour	1	2	3	4
AE	In this community many people think that a woman must continue to have children until she has a son	1	2	3	4
AF	I think that a wife in my family must continue to have children until she has a son	1	2	3	4
AG	In this community many people think that its men's responsibility to control the behavior of women in their family to protect the family from gossip	1	2	3	4
AH	I think that the men in my family have the responsibility of controlling the behavior of	1	2	3	4

	women in our family to protect the family from gossip				
AI	In this community many people think that a man should have the final say in all family matters	1	2	3	4
AJ	I think that men in my family should have the final say in all family matters	1	2	3	4
AK	In this community many people that a man should have the final say in all family matters	1	2	3	4
AL	I think that men in my family should have the final say in all family matters	1	2	3	4
AM	In this community many people think that a woman cannot refuse to have sex with her husband	1	2	3	4
AN	I think that a wife in the family cannot refuse to have sex with her husband.	1	2	3	4
AO	In this community many people think that there is nothing a woman can do if her husband wants to take a second wife	1	2	3	4
AP	I think that there is nothing a wife in the family can do if her husband wants to take a second wife	1	2	3	4
AQ	In this community many people think that men should share the work around the house with their wife such as doing dishes, cleaning and cooking	1	2	3	4
AR	I think that men in our family should share the work around the house with their wife such as doing dishes, cleaning and cooking	1	2	3	4
AS	In this community many people think that if your husband beats his wife it shows that he loves her	1	2	3	4
AT	I think that if men in our family beats their wives it shows that they loves them.	1	2	3	4

SECTION 3 YOUR HEALTH

NO.	QUESTIONS	CODING CATEGORIES				SKIP TO
The next questions we would like to ask are about how you have been feeling in the <u>past week</u>. Each question is a statement; please answer how many days you have had particular feelings or ideas or whether you have not had them at all. There are four options: rarely or never, one 1-2 days, on 3-4 days, or 5-7 days (most or all of the time).						
301	**CES-D SCALE**	RARELY OR NONE OF THE TIME	SOME OR A LITTLE OF THE TIME (1-2 DAYS)	MODERATE AMOUNT OF TIME (3-4 DAYS)	MOST OR ALL OF THE TIME (5-7 DAYS)	
**A**	During the past week I was bothered by things that usually don't bother me	**0**	**1**	**2**	**3**	
**B**	During the past week I did not feel like eating, my appetite was poor	**0**	**1**	**2**	**3**	
**C**	During the past week I felt I could not cheer myself up even with the help of family and friends	**0**	**1**	**2**	**3**	

**D**	During the past week I felt I was just as good as other people	**0**	**1**	**2**	**3**	
**E**	During the past week I had trouble keeping my mind on what I was doing	**0**	**1**	**2**	**3**	
**F**	During the past week I felt depressed	**0**	**1**	**2**	**3**	
**G**	During the past week I felt that everything I did was an effort	**0**	**1**	**2**	**3**	
**H**	During the past week I felt hopeful about the future	**0**	**1**	**2**	**3**	
**I**	During the past week I thought my life had been a failure	**0**	**1**	**2**	**3**	
**J**	During the past week I felt fearful	**0**	**1**	**2**	**3**	
**K**	During the past week my sleep was restless	**0**	**1**	**2**	**3**	
**L**	During the past week I was happy	**0**	**1**	**2**	**3**	
**M**	During the past week I talked less than usual	**0**	**1**	**2**	**3**	
**N**	During the past week I felt lonely	**0**	**1**	**2**	**3**	
**O**	During the past week people were unfriendly	**0**	**1**	**2**	**3**	
**P**	During the past week I enjoyed life	**0**	**1**	**2**	**3**	
**Q**	During the past week I had crying spells	**0**	**1**	**2**	**3**	
**R**	During the past week I felt sick	**0**	**1**	**2**	**3**	
**S**	During the past week I felt that people dislike me	**0**	**1**	**2**	**3**	
**T**	During the past week I could not get 'going'	**0**	**1**	**2**	**3**	

SECTION 4 HER HUSBAND AND HOME

You are progressing very well, thank you. Now we have some questions about your husband (or most recent husband)

401	How old is your husband?	AGE (YEARS) ....[ ][ ]				
402	I want to ask you some questions about what sort of man he is. Please answer if you strongly agree, agree, disagree or strongly disagree with each of these statements	*Strongly disagree* 1	*Disagree* 2	*Agree* 3	*Strongly agree* 4	
A	My husband does not really understand me.	1	2	3	4	
B	My husband does everything he can to support me.	1	2	3	4	
C	My husband is a kind person.	1	2	3	4	

D	My husband is too busy to spend much time with me.	1	2	3	4	
E	My husband is very strict and controlling.	1	2	3	4	
F	My husband can be cruel	1	2	3	4	
G	My husband can frighten me	1	2	3	4	
405	I want to ask you some questions about your mother-in-law. Please answer if you strongly agree, agree, disagree or strongly disagree with each of these statements	*Strongly disagree* 1	*Disagree* 2	*Agree* 3	*Strongly agree* 4	
A	My mother-in-law does not really understand me.	1	2	3	4	
B	My mother-in-law does everything she can to support me.	1	2	3	4	
C	My mother-in-law is a kind person.	1	2	3	4	
D	My mother-in-law loves me like her own daughter	1	2	3	4	
E	My mother-in-law is very strict and controlling.	1	2	3	4	
F	My mother-in-law can be cruel	1	2	3	4	
G	My mother-in-law can frighten me	1	2	3	4	
406	In the last three months, how often have your views been listened to on your health or that of other women and children in your home?	NEVER…………………………………………..1<br>SOMETIMES……………………………………2<br>OFTEN…………………………………………. 3				
407	In the last three months, how often your views been listened to on matters concerning the children and their schooling or work in your home?	NEVER…………………………………………..1<br>SOMETIMES……………………………………2<br>OFTEN…………………………………………. 3<br>NO CHILDREN…………………………………4				
408	In the last three months, how often have your views been listened to on problems which your husband or family faces?	NEVER…………………………………………..1<br>SOMETIMES……………………………………2<br>OFTEN…………………………………………. 3				
409	In the last three months, how often have your views been listened to on decisions to buy large items such as livestock or land or a television or air conditioner?	NEVER…………………………………………..1<br>SOMETIMES……………………………………2<br>OFTEN…………………………………………. 3<br>WE HAVE NOT DISCUSSED BUYING LARGE ITEMS…………………………………………..4				
410	In the last three months, how often have your views been listened to on when you or other women in the family can go to events in the village or join groups?	NEVER…………………………………………..1<br>SOMETIMES……………………………………2<br>OFTEN…………………………………………. 3				

The next set of statements are about your relationship with your **husband**, please say for each if you strongly agree, agree, disagree or strongly disagree:

417	RELATIONSHIP CONTROL SCALE	STRONGLY DISAGREE	DISAGREE	AGREE	STRONGLY AGREE
A	When he wants sex he expects me to agree	1	2	3	4
B	He won't let me spend money on things for myself	1	2	3	4
C	He won't let me wear certain things	1	2	3	4

D	He won’t let me have a mobile phone	1	2	3	4
E	He tells me who I can spend time with.	1	2	3	4
F	When I wears things to make me look beautiful he thinks I may be trying to attract other men	1	2	3	4
G	He wants to know where I am all of the time	1	2	3	4
H	He lets me know he could always take another wife	1	2	3	4

In any marriage there are good times and bad times, I now want to ask you about some of the bad times and what has happened. Remember there are no right or wrong answers and everything you say will be kept secret.					
		NEVER	ONCE	FEW	MANY
418	In the past 12 months how often did your husband stop you from getting a job, going to work, trading or earning money?	0	1	2	3
419	In the past 12 months how often did your husband take your earnings against your will?	0	1	2	3
420	In the past 12 months how often did your husband throw you out of the house?	0	1	2	3
421	In the past 12 months how often did your husband spend money on alcohol, tobacco or other things for himself when he knew you did not have enough for essential household expenses?	0	1	2	3
422	In the past 12 months how many times has your husband insulted you or made you feel bad about yourself?	0	1	2	3
423	In the past 12 months how many times has your husband belittled or humiliated you in front of other people?	0	1	2	3
424	In the past 12 months how many times has your husband done things to scare or intimidate you on purpose for example, by the way he looked at you, by yelling or smashing things?	0	1	2	3
425	In the past 12 months how many times has your husband threatened to hurt you?	0	1	2	3
426	In the past 12 months how many times has your husband threatened to divorce you?	0	1	2	3
427	In the past 12 months how many times has your husband hurt people you care about as a way of hurting you, or damaged things of importance to you?	0	1	2	3
428	In the past 12 months how many times has your partner spend money on things for himself when he knew there was not enough money for food or school fees or other essential household expenses?	0	1	2	3
429	In the past 12 months how many times has your husband slapped you or thrown something at you which could hurt you?	0	1	2	3
430	In the past 12 months how many times has your husband pushed or shoved you?	0	1	2	3
431	In the past 12 months how many times has your husband hit you with a fist or with something else which could hurt you?	0	1	2	3
432	In the past 12 months, how many times has your husband kicked, dragged, beaten, choked or burnt you?	0	1	2	3
433	In the past 12 months, how many times has your husband threatened to use or actually used a gun, knife or other weapon against you?	0	1	2	3
		NEVER	ONCE	FEW	MANY

434	In the past 12 months, how many times has a current or previous husband or boyfriend ever physically forced you to have sex when you did not want to?	0	1	2	3
435	In the past 12 months, how many times has your current or previous boyfriend, husband or partner used threats or intimidation to get you to have sex when you did not want to?	0	1	2	3
436	In the past 12 months, how many times has a current or previous husband or boyfriend ever forced you to do something else sexual that did not want to do?	0	1	2	3

Thank you for answering these questions. Please remember no one will know that you have told us these things. I would like to now ask the same questions about any time in your life.

		NEVER	ONCE	FEW	MANY
437	How many times has your current or a previous husband ever slapped you or thrown something at you which could hurt you?	0	1	2	3
438	How many times has your current or a previous husband ever pushed or shoved you?	0	1	2	3
439	How many times has your current or a previous husband ever hit you with a fist or with something else which could hurt you?	0	1	2	3
440	How many times has your current or a previous husband ever kicked, dragged, beaten, choked or burnt you?	0	1	2	3
441	How many times has your current or a previous husband ever threatened to use or actually used a gun, knife or other weapon against you?	0	1	2	3
		**NEVER**	**ONCE**	**FEW**	**MANY**
442	How many times has a current or previous husband ever physically forced you to have sex when you did not want to?	0	1	2	3
443	How many times has a current or previous husband ever used threats or intimidation to get you to have sex when you did not want to?	0	1	2	3
444	How many times did a current or previous husband ever force you to do something else sexual that you did not want to do?	0	1	2	3

	**SECTION 5: CHILDHOOD AND OTHER LIFE EXPERIENCES**				
	**THESE QUESTIONS ARE PHRASED AS "BEFORE YOU WERE MARRIED" BUT IF THE WOMAN HAS NOT BEEN MARRIED, PLEASE REPHRASE EACH AS "BEFORE YOU WERE 18…"**				
	Thank you for answering these questions. The questionnaire will be finished soon. We would just like to ask you some questions about **your childhood before you married** and other experiences you have had in your life. First we have a series of statements about your childhood. For each we would like to know if they never, sometimes, often or very often happened				
501		NEVER	SOMETIMES	OFTEN	VERY OFTEN
A	Before I married I did not have enough to eat	1	2	3	4
B	Before I married I lived in different households at different times	1	2	3	4

C	Before I married I saw or heard my mother being beaten by her husband	1	2	3	4
D	Before I married I was told I was lazy or stupid or weak by someone in my family	1	2	3	4
E	Before I married I saw or heard my mother being beaten by my mother-in-law or another person in the family	1	2	3	4
F	Before I married I was insulted or humiliated by someone in my family in front of other people	1	2	3	4
G	Before I married I was beaten at home with a belt or stick or whip or something else which was hard	1	2	3	4
H	Before I married I had to work at home to help the family get money	1	2	3	4
I	Before I married one or both of my parents was not able to take care of me	1	2	3	4
J	Before I married I was beaten so hard at home that it left a mark or injured me	1	2	3	4
K	Before I married I was able to spend time outside the home in fields or in the garden or orchard	1	2	3	4
L	Before I married I was often afraid we would be killed or injured in the conflict or war	1	2	3	4

THANK YOU for completing this interview.
