# Supplementary material for: Factors associated with IPV victimisation of women and perpetration by men in migrant communities of Nepal
Source: PLoS One. 2019 Jul 30;14(7):e0210258. doi: 10.1371/journal.pone.0210258 (PMC6667197; doi:10.1371/journal.pone.0210258)
Supplement: S6 Table — (PDF) [file pone.0210258.s006.pdf]

MEN’S

QUESTIONNAIRE

BASELINE

COVER

Study ID number: _______________

DATE: ________________

SECTION ONE: BACKGROUND & HOME ***The first questions are about yourself, your home and your work situation. Please try and relax, there are no right or wrong answers. Remember that everything you answer will be kept secret.***			
**QUESTIONS AND FILTERS**		**CODING CATEGORIES**	
101	How old are you?	AGE (YEARS)	
102	What is your ethnic group?	Dalit ....1<br>Janjati ....2<br>Chettri ....3<br>Brahman..4<br>Other 5	
104	Did you attend school?	Yes....................................1<br>No.....................................0	If no<br>→ 108
105	What is your highest level of schooling?	Primary (1-4) ....1<br>Completed primary (5)......................................2<br>Lower secondary (6-8).....................................3<br>Secondary (9) ....4<br>Completed secondary (10) ....5<br>SLC ....6<br>Intermediate/10+2 ....7<br>Graduate/Bachelors ....8 Post<br>Graduate/Masters & PhD...........................9	
109	Are you current married or have you been previously married?	CURRENTLY MARRIED..............................1<br>PREVIOUSLY MARRIED...........................2<br>NEVER MARRIED..3	If 1=>110<br><br>If 3, no<br>→ 116
110	How long have you been married? (Or how long were you married for?)	[ ] YEARS	
114	Do you live (READ OPTIONS):	ALONE WITH YOUR WIFE AND CHILDREN (IF YOU HAVE CHILDREN) ....1<br>IN SAME HOUSEHOLD WITH YOUR WIFE'S FAMILY..................................2<br>IN SAME HOUSEHOLD WITH YOUR WIFE AND YOUR OWN FAMILY......................3<br>ALONE WITH YOUR CHILDREN...................4<br>WITH YOUR FAMILY.......................................5<br>ALONE...6	

WORK AND MONEY			
116	In the past 4 weeks, how often was there no food to eat of any kind in your house because of a lack of money?	Often……………………………………………………….4<br>Sometimes…………………………………………………3<br>Rarely……………………………………………………….2<br>Never……………………………………………………….1	
117	In the past 4 weeks how often did you or any member of your household go to sleep hungry because of lack of food?	Often……………………………………………………….4<br>Sometimes…………………………………………………3<br>Rarely……………………………………………………….2<br>Never……………………………………………………….1	
118	In the past 4 weeks how often did people in your home go without meat due to lack or money or to save money?	OFTEN HAVE NO MEAT……………………………………1<br>SOMETIMES HAVE NO MEAT……………………………2<br>OCCCASIONALLY HAVE NO MEAT…………………3<br>ALWAYS HAS MEAT…………………………………………4<br>VEGETARIAN………………………………………………….5	
119	In the past 4 weeks how often did people in your home was eating fruit like apple, orange, mango, papaya.	EVERY DAY……………………………………………………5<br>MORE THAN ONCE A WEEK………………………………4<br>ALMOST EVERY WEEK………………………………………3<br>ONCE OR TWICE IN THE LAST 4 WEEKS……………2<br>NEVER………………………………………………………….1	
120	In the past 4 weeks how often did you or any of your household go a whole day and night without eating because of lack of food?	Often……………………………………………………….4<br>Sometimes…………………………………………………3<br>Rarely……………………………………………………….2<br>Never……………………………………………………….1	
121	If you had an emergency at home and needed 1500 Nepal Rupees, how easy would you say it would be to find the money?	VERY DIFFICULT……………………………………………1<br>SOMEWHAT DIFFICULT……………………………………2<br>FAIRLY EASY…………………………………………………3<br>VERY EASY……………………………………………………4	
122	How often in the past 4 weeks have you had to borrow food or money because you did not have enough?	EVERYDAY……………………………………………………5<br>MORE THAN ONCE A WEEK………………………………4<br>ALMOST EVERY WEEK………………………………………3<br>ONCE OR TWICE IN THE LAST 4 WEEKS……………2<br>NEVER………………………………………………………….1	
123	Have you done anything which has earned money for you or your family in the last 3 months?	YES…………………………………………………………………1<br>NO…………………………………………………………………0	IF NO<br>➔126

127	Have you ever moved away from home in for work?	YES……………………………………………1<br>NO………………………………………………0			IF NO →132
129	In the last 12 months have you worked away from home?	YES……………………………………………1<br>NO………………………………………………0			IF NO →132
132	For the following statements, please tell me how often in the **last 3 months** you have done the following:	NEVER	ONCE	A FEW TIMES	MANY TIMES
	Searched for work	1	2	3	4
	Searched newspapers for jobs	1	2	3	4
	Handed in or sent off an application for work	1	2	3	4
	Offered to work without pay to get experience	1	2	3	4
	Worked without pay to get experience	1	2	3	4
	Developed an idea for a way of earning by selling or making things	1	2	3	4
	Earned money through selling or making things	1	2	3	4
133	The following statements are a series of statements about your current work situation. Please say if you strongly agree, agree, disagree or strongly disagree with these phrases:	STRONGLY DISAGREE	DISAGREE	AGREE	STRONGLY AGREE
	I am frequently stressed or depressed because of not having enough work	1	2	3	4
	I am frequently stressed or depressed because of not having enough income	1	2	3	4
	I am frequently stressed or depressed because I am not proud of what I do to get money	1	2	3	4
	I am frequently stressed or depressed because I want or have to help my family with money	1	2	3	4

134	The following statements are series of statements about your work situation. Please say if you strongly agree, agree, disagree or strongly disagree with these phrases:	STRONGLY DISAGREE	DISAGREE	AGREE	STRONGLY AGREE
	I sometimes feel ashamed to face my family because I am out of work.	1	2	3	4
	I spend most of my time out of work or looking for work	1	2	3	4
	I have given up looking for work because I never find any	1	2	3	4
	I am ashamed to see my wife and children because I don't have money	1	2	3	4

SECTION 2 ATTITUDES ABOUT RELATIONS BETWEEN MEN AND WOMEN

The next set of questions are about your views on life and particularly on relations between men and women in society. There are no right or wrong answers – we are just interested in what you think.

For each of the following statements please say answer whether you strongly agree, agree, disagree or strongly disagree with the following statements:

201		STRONGLY DISAGREE	DISAGREE	AGREE	STRONGLY AGREE
A	In this community most people think that girls should complete secondary school (to grade 10)	1	2	3	4
B	I think girls in my family should go complete secondary school (to grade 10)	1	2	3	4
C	In this community most people think that girls should continue their education after completing secondary school	1	2	3	4
D	I think girls in my family should continue their education after completing secondary school	1	2	3	4
E	In this community many people think that a wife must ask permission from her husband or his family before going somewhere	1	2	3	4
F	I think the husbands in my family must ask permission from her husband or his family before going somewhere	1	2	3	4
G	In this community many people think that wives who live with their husband's parents should have a say in how money is spent	1	2	3	4
H	I think the wives who live with their husband's parents should have a say in how money is spent	1	2	3	4
I	In this community many people think that husbands should allow their wives to do something at home to generate income for the family	1	2	3	4
J	I think the husbands in my family should allow their wives to do something at home to generate income for the family	1	2	3	4
K	In this community many people think that husbands should allow their wives to do something outside the home to generate income for the family	1	2	3	4
L	I think the husbands in my family should allow their wives to do something outside the home to generate income for the family	1	2	3	4

M	In this community many people think that husbands should allow their wives to have a job to contribute to the family budget	1	2	3	4
N	I think the husbands in my family should allow their wives to have a job to contribute to the family budget	1	2	3	4
O	In this community many people think that a husband should be kind and care about the happiness of women in his family	1	2	3	4
P	I think husbands in my family should be kind and care about the happiness of women in the family	1	2	3	4
Q	In this community many people think that wives must always obey their husband	1	2	3	4
R	I think that the wives in my family must always obey their husbands	1	2	3	4
S	In this community many people think that a daughter-in-law must always obey her mother-in-law	1	2	3	4
T	I think that the daughters-in-law in my family must always obey their mother-in-law	1	2	3	4
U	In this community many people think that if a wife does something wrong her husband has the right to punish her	1	2	3	4
V	I think that if a wife in my family does something wrong her husband has the right to punish her	1	2	3	4
W	In this community many people think that a wife of any age who does things that are wrong should be beaten to correct her behavior	1	2	3	4
X	I think that a wife in my family of any age who does things that are wrong should be beaten to correct her behavior	1	2	3	4
Y	In this community many people think that it is acceptable to beat a young wife to teach her how to behave properly	1	2	3	4
Z	I think it is acceptable for a young wife in my family to be beaten to teach her how to behave properly	1	2	3	4
AA	In this community many people think that a woman should tolerate violence in order to keep her family together	1	2	3	4
AB	I think that a wife in my family should tolerate violence in order to keep her family together	1	2	3	4
AC	In this community many people think that a woman should tolerate violence for sake of family honour	1	2	3	4
AD	I think that a wife in my family should tolerate violence for sake of family honour	1	2	3	4
AE	In this community many people think that a woman must continue to have children until she has a son	1	2	3	4
AF	I think that a wife in my family must continue to have children until she has a son	1	2	3	4

AG	In this community many people think that its men's responsibility to control the behavior of women in their family to protect the family from gossip	1	2	3	4
AH	I think that the men in my family have the responsibility of controlling the behavior of women in our family to protect the family from gossip	1	2	3	4
AI	In this community many people think that a man should have the final say in all family matters	1	2	3	4
AJ	I think that men in my family should have the final say in all family matters	1	2	3	4
AK	In this community many people that a man should have the final say in all family matters	1	2	3	4
AL	I think that men in my family should have the final say in all family matters	1	2	3	4
AM	In this community many people think that a woman cannot refuse to have sex with her husband	1	2	3	4
AN	I think that a wife in the family cannot refuse to have sex with her husband.	1	2	3	4
AO	In this community many people think that there is nothing a woman can do if her husband wants to take a second wife	1	2	3	4
AP	I think that there is nothing a wife in the family can do if her husband wants to take a second wife	1	2	3	4
AQ	In this community many people think that men should share the work around the house with their wife such as doing dishes, cleaning and cooking	1	2	3	4
AR	I think that men in our family should share the work around the house with their wife such as doing dishes, cleaning and cooking	1	2	3	4
AS	In this community many people think that if your husband beats his wife it shows that he loves her	1	2	3	4
AT	I think that if men in our family beats their wives it shows that they love them	1	2	3	4
AU	If someone insults a man, he should defend his reputation, with force if he has to	1	2	3	4
AV	To be a man, a person needs to be tough	1	2	3	4

SECTION 3 YOUR HEALTH

NO.	QUESTIONS	CODING CATEGORIES				SKIP TO
	The next questions we would like to ask are about how you have been feeling in the past week. Each question is a statement; please answer how many days you have had particular feelings or ideas or whether you have not had them at all. There are four options: rarely or never, one 1-2 days, on 304 days, or 5-7 days (most or all of the time).					
301	**CES-D SCALE**	RARELY OR NONE OF THE TIME	SOME OR A LITTLE OF THE TIME (1-2 DAYS)	MODERATE AMOUNT OF TIME (3-4 DAYS)	MOST OR ALL OF THE TIME (5-7 DAYS)	
**A**	During the past week I was bothered by things that usually don't bother me	**0**	**1**	**2**	**3**	
**B**	During the past week I did not feel like eating, my appetite was poor	**0**	**1**	**2**	**3**	
**C**	During the past week I felt I could not cheer myself up even with the help of family and friends	**0**	**1**	**2**	**3**	
**D**	During the past week I felt I was just as good as other people	**0**	**1**	**2**	**3**	
**E**	During the past week I had trouble keeping my mind on what I was doing	**0**	**1**	**2**	**3**	
**F**	During the past week I felt depressed	**0**	**1**	**2**	**3**	
**G**	During the past week I felt that everything I did was an effort	**0**	**1**	**2**	**3**	
**H**	During the past week I felt hopeful about the future	**0**	**1**	**2**	**3**	
**I**	During the past week I thought my life had been a failure	**0**	**1**	**2**	**3**	
**J**	During the past week I felt fearful	**0**	**1**	**2**	**3**	
**K**	During the past week my sleep was restless	**0**	**1**	**2**	**3**	
**L**	During the past week I was happy	**0**	**1**	**2**	**3**	

**M**	During the past week I talked less than usual	**0**	**1**	**2**	**3**	
**N**	During the past week I felt lonely	**0**	**1**	**2**	**3**	
**O**	During the past week people were unfriendly	**0**	**1**	**2**	**3**	
**P**	During the past week I enjoyed life	**0**	**1**	**2**	**3**	
**Q**	During the past week I had crying spells	**0**	**1**	**2**	**3**	
**R**	During the past week I felt sick	**0**	**1**	**2**	**3**	
**S**	During the past week I felt that people dislike me	**0**	**1**	**2**	**3**	
**T**	During the past week I could not get 'going'	**0**	**1**	**2**	**3**	

SECTION 4 HIS WIFE AND HOME

You are progressing very well, thank you. Now we have some questions about your wife (or most recent wife)						
401	How old is your wife?	AGE (YEARS) ....[ ][ ]				
402	I want to ask you some questions about what sort of woman she is. Please answer if you strongly agree, agree, disagree or strongly disagree with each of these statements	STRONGLY DISAGREE	DISAGREE	AGREE	STRONGLY AGREE	
A	My wife does not really understand me.	1	2	3	4	
B	My wife does everything she can to support me.	1	2	3	4	
C	My wife is a kind person.	1	2	3	4	

D	My wife works too much in the household.	1	2	3	4	
E	My wife spends too much time outside of the home	1	2	3	4	
F	I can trust my wife with money	1	2	3	4	
G	My wife understands how hard I work	1	2	3	4	
405	I want to ask you some questions about your mother. Please answer if you strongly agree, agree, disagree or strongly disagree with each of these statements	*Strongly disagree* 1	*Disagree* 2	*Agree* 3	*Strongly agree* 4	
A	My mother does not really understand me.	1	2	3	4	
B	My mother does everything she can to support me.	1	2	3	4	
C	My mother is a kind person.	1	2	3	4	
D	My mother loves my wife like her own daughter	1	2	3	4	
E	My mother is very strict and controlling.	1	2	3	4	
F	My mother can be cruel.	1	2	3	4	
G	My mother can frighten me.	1	2	3	4	
406	In the last three months, how often have your views been listened to on your health or that of children in your home?	NEVER…………………………………………1<br>SOMETIMES……………………………………2<br>OFTEN………………………………………… 3				
407	In the last three months, how often your views been listened to on matters concerning the children and their schooling or work?	NEVER…………………………………………1<br>SOMETIMES……………………………………2<br>OFTEN………………………………………… 3<br>NO CHILDREN…………………………………4				

408	In the last three months, how often have your views been listened to on problems which your wife or family faces?	NEVER...1<br>SOMETIMES..2<br>OFTEN.. 3				
409	In the last three months, how often have your views been listened to on decisions to buy large items such as livestock or land or a television or air conditioner?	NEVER...1<br>SOMETIMES..2<br>OFTEN.. 3<br>WE HAVE NOT DISCUSSED BUYING LARGE ITEMS..4				
	The next set of statements are about your relationship with your **wife**, please say for each if you strongly agree, agree, disagree or strongly disagree:					
417	**RELATIONSHIP CONTROL SCALE**	STRONGLY DISAGREE	DISAGREE	AGREE	STRONGLY AGREE	
A	When I want sex I expect her to agree	1	2	3	4	
B	I won't let her spend money on things for myself	1	2	3	4	
C	I won't let her wear certain things	1	2	3	4	
D	I won't let her have a mobile phone	1	2	3	4	
E	I tell her who she can spend time with.	1	2	3	4	
F	When my wife wears things to make her look beautiful I think she may be trying to attract other men	1	2	3	4	
G	I want to know where my wife is all of the time.	1	2	3	4	
H	I like to let her know she isn't the only wife I could have.	1	2	3	4	

In any marriage there are good times and bad times, I now want to ask you about some of the bad times and what has happened. Remember there are no right or wrong answers and everything you say will be kept secret.					
		**NEVER**	**ONCE**	**FEW**	**MANY**
418	In the past 12 months how often did you stop your wife from getting a job, going to work, trading or earning money?	0	1	2	3
419	In the past 12 months how often did you take your wife's earnings against her will?	0	1	2	3
420	In the past 12 months how often did you throw your wife out of the house?	0	1	2	3

421	In the past 12 months how often did spend money on alcohol, tobacco or other things when you knew your wife did not have enough for essential household expenses?	0	1	2	3
422	In the past 12 months how many times have you insulted your wife or made her feel bad about herself?	0	1	2	3
423	In the past 12 months how many times have you belittled or humiliated her in front of other people?	0	1	2	3
424	In the past 12 months how many times have you done things to scare or intimidate her on purpose for example, by the way you looked at her, by yelling or smashing things?	0	1	2	3
425	In the past 12 months how many times have you threatened to hurt her?	0	1	2	3
426	In the past 12 months how many times have you threatened to divorce her?	0	1	2	3
427	In the past 12 months how many times have you hurt people she cares about as a way of hurting her, or damaged things of importance to her?	0	1	2	3
428	In the past 12 months how many times have you spent money on things for yourself when you knew there was not enough money for food or school fees or other essential household expenses?	0	1	2	3
429	In the past 12 months how many times have you slapped your wife or thrown something at you which could hurt you?	0	1	2	3
430	In the past 12 months how many times have you pushed or shoved her?	0	1	2	3
431	In the past 12 months how many times have you hit her with a fist or with something else which could hurt her?	0	1	2	3
432	In the past 12 months, how many times have you kicked, dragged, beaten, choked or burnt her?	0	1	2	3
433	In the past 12 months, how many times have you threatened to use or actually used a gun, knife or other weapon against her?	0	1	2	3
		**NEVER**	**ONCE**	**FEW**	**MANY**
434	In the past 12 months, how many times have you ever physically forced your wife or a girlfriend to have sex when she did not want to?	0	1	2	3
435	In the past 12 months, how many times have you used threats or intimidation to get your wife or a girlfriend to have sex when she did not want to?	0	1	2	3

436	In the past 12 months, how many times have you ever forced your wife or a girlfriend to do something else sexual that did not want to do?	0	1	2	3

Thank you for answering these questions. Please remember no one will know that you have told us these things. I would like to now ask the same questions about any time in your life.					
		**NEVER**	**ONCE**	**FEW**	**MANY**
437	How many times have you ever slapped you or thrown something at your wife or a girlfriend which could hurt her?	0	1	2	3
438	How many times have you ever pushed or shoved your wife or a girlfriend?	0	1	2	3
439	How many times has your current have you ever hit you with a fist or with something else which could hurt your wife or a girlfriend?	0	1	2	3
440	How many times have you ever kicked, dragged, beaten, choked or burnt your wife or a girlfriend?	0	1	2	3
441	How many times have you ever threatened to use or actually used a gun, knife or other weapon against your wife or a girlfriend?	0	1	2	3
		**NEVER**	**ONCE**	**FEW**	**MANY**
442	How many times have you ever physically forced your wife or a girlfriend to have sex when your wife or a girlfriend did not want to?	0	1	2	3
443	How many times have you ever used threats or intimidation to get your wife or a girlfriend to have sex when your wife or a girlfriend did not want to?	0	1	2	3
444	How many times did you ever force your wife or a girlfriend to do something else sexual that she did not want to do?	0	1	2	3

	**SECTION 5: CHILDHOOD AND OTHER LIFE EXPERIENCES**
	**THESE QUESTIONS ARE PHRASED AS "BEFORE YOU WERE MARRIED" BUT IF THE WOMAN HAS NOT BEEN MARRIED, PLEASE REPHRASE EACH AS "BEFORE YOU WERE 18…"**

	Thank you for answering these questions. The questionnaire will be finished soon. We would just like to ask you some questions about **your childhood before you married** and other experiences you have had in your life. First we have a series of statements about your childhood. For each we would like to know if they never, sometimes, often or very often happened				
501		NEVER	SOMETIMES	OFTEN	VERY OFTEN
A	Before I married I did not have enough to eat	1	2	3	4
B	Before I married I lived in different households at different times	1	2	3	4
C	Before I married I saw or heard my mother being beaten by her husband	1	2	3	4
D	Before I married I was told I was lazy or stupid or weak by someone in my family	1	2	3	4
E	Before I married I saw or heard my mother being beaten by my mother-in-law or another person in the family	1	2	3	4
F	Before I married I was insulted or humiliated by someone in my family in front of other people	1	2	3	4
G	Before I married I was beaten at home with a belt or stick or whip or something else which was hard	1	2	3	4
H	Before I married I had to work at home to help the family get money	1	2	3	4
I	Before I married one or both of my parents was not able to take care of me	1	2	3	4
J	Before I married I was beaten so hard at home that it left a mark or injured me	1	2	3	4
K	Before I married I was able to spend time outside the home in fields or in the garden or orchard	1	2	3	4
L	Before I married I was often afraid we would be killed or injured in the conflict or war	1	2	3	4

THANK YOU for completing this interview.
