# Supplementary material for: Factors associated with IPV victimisation of women and perpetration by men in migrant communities of Nepal
Source: PLoS One. 2019 Jul 30;14(7):e0210258. doi: 10.1371/journal.pone.0210258 (PMC6667197; doi:10.1371/journal.pone.0210258)
Supplement: S7 Table — (PDF) [file pone.0210258.s007.pdf]

LIST OF DERIVED VARIABLES

variable name	variable label	value labels
age	Age of participant	
ethnic	Ethnicity	1=Dalit; 2=Janjati; 3=Chhetri; 4=Brahmin; 5=other
educ	Level of education passed	0=no education; 1=Primary; 2=Secondary; 3=SLC plus(school leaving)
marital	Marital status	1=married; 2=previously married; 3=never married
yrsmarried	Years married	
living_arrangement	current living arrangements	1=with partner & children; 2=with partner's family; 3=with partner and natal family; 4=alone with children; 5=with natal family
older	Type of participant	1=young 2=older
relcon	relationship control (score) high=more control	
chtr	Childhood trauma (score) high=more trauma	
wksk	Work stress (score) high=more stress	
CommNorms	Community gender attitudes (score) high=patriarchal	
husrel	women's relations with husband (score)	
wfrel	husband's relation with wife (score)	
genattsI	individual gender attitudes (score) high = patriarchal	
unempstr	unemployment stress (score)	
workshame	shame due to lack of work (score)	
MILcruel	mother/mother-in-law cruelty(score)	
MILkind	mother/mother-in-law kindness (score)	
womdec	woman's involvement in decision-making , excluded women with no children (score)	
mandec	man's involvement in decision-making, excluded men with no children (score)	
difmony	Difficult to get money 1=yes	0=no 1=yes
borroweva	Have borrowed money ever 1=yes	0=no 1=yes
migratework	Ever migrated for work 1=yes	0=no 1=yes
migratework12m	Migrated for work in last 12m 1=yes	0=no 1=yes
incomegeneration	Engaged in an activity to generate income	0=no 1=yes
lifsat	life dissatisfaction higher=more dissatisfied (score)	
cesd	Depression high=more depressed (score)	
cesdep	Depressed 1=yes	0=no 1=yes
hope	Hope high=more hopeful (score)	
hung	hunger score, high= hungrier	
pipv12	any physical ipv in past 12m 1=yes	0=no 1=yes
sipv12	any s ipv past 12m	0=no 1=yes
emab12	any emotional ipv in last 12m	0=no 1=yes
pipv	any physical ipv ever 1=yes	0=no 1=yes
sipv	any sexual ipv ever	0=no 1=yes
psipv	phyiscal or sexual IPV ever	0=no 1=yes

empsipv12	any emotional,sexual or physical ipv in last 12m	0=no 1=yes
health	Self assessment of health 1=good/excellent	0=no 1=good/excellent
health2	Self assessment of health 1=fairly good/excellent	0=no 1=fairly good/excellent
suicidal	Have suicidal thoughts or attempted suicide 1=yes	0=no 1=yes
quarrel	Frequency of quarrelling 1=sometimes/often	0=none 1=sometimes/often
alcohol	Ever drunk alcohol	0=no 1=yes
alcohol12m	Drunk alcohol in past 12m	0=no 1=yes
partdrink	Partner drinks alcohol 1=yes	0=no 1=yes
partnerage	Partner's age	
ageatfirstmarriage	Age at first marriage	
